# Cardiometabolic Health in Pregnancy and Postpartum: Findings From a Prospective Cohort Study in South Africa

**DOI:** 10.1093/ofid/ofae093

**Published:** 2024-02-22

**Authors:** Angela M Bengtson, Hlengiwe Madlala, Mushi J Matjila, Julia H Goedecke, Susan Cu-Uvin, Stephen T McGarvey, Erika Werner, Landon Myer

**Affiliations:** Department of Epidemiology, Emory University, Atlanta, Georgia, USA; Division of Epidemiology and Biostatistics, School of Public Health, University of Cape Town, Cape Town, South Africa; Department of Obstetrics & Gynaecology, University of Cape Town, Groote Schuur and New Somerset Hospitals, Cape Town, South Africa; Biomedical Research and Innovation Platform, South African Medical Research Council, Cape Town, South Africa; Health through Physical Activity, Lifestyle and Sport Research Centre, Division of Physiological Sciences, Department of Human Biology, University of Cape Town, Cape Town, South Africa; Department of Obstetrics and Gynecology and Medicine, Warren Alpert School of Medicine, Brown University, Providence, Rhode Island, USA; Department of Epidemiology and International Health Institute, Brown University School of Public Health, Providence, Rhode Island, USA; Department of Obstetrics and Gynecology, Tufts University School of Medicine, Boston, Massachusetts, USA; Division of Epidemiology and Biostatistics, School of Public Health, University of Cape Town, Cape Town, South Africa

**Keywords:** antiretroviral therapy, blood pressure, cardiometabolic health, dolutegravir, HIV

## Abstract

**Background:**

The cardiometabolic impact of HIV infection and treatment with antiretroviral therapy (ART) in pregnancy and the postpartum period remains unclear.

**Methods:**

We enrolled pregnant persons with (PHIV) and without HIV in Cape Town, South Africa, who were ≥18 years old at 24–28 weeks’ gestation and followed them up to 32 months postpartum. We estimated associations between HIV status and cardiometabolic risk including body mass index (BMI), obesity (BMI ≥30 kg/m^2^), blood pressure (BP; elevated systolic BP ≥130 and/or diastolic ≥85 mmHg), lipid levels, and metabolic syndrome according to the Joint Interim Statement criteria using multivariable log binomial or linear regression models. Subgroup analyses compared PHIV on efavirenz (EFV)- vs dolutegravir (DTG)-based ART.

**Results:**

Among 400 participants (n = 200 without HIV, n = 200 PHIV), 52% had prepregnancy obesity and 9% had elevated BP. Postpartum, 57% were classified with obesity, 31% had elevated BP, and 29% had metabolic syndrome. In multivariable analyses, HIV was associated with a lower BMI prepregnancy but not postpartum; however, mean indices were in the obese range regardless of HIV status. Neither BMI nor obesity prepregnancy or postpartum differed by ART regimen. Among PHIV, participants on DTG had higher levels of elevated BP in pregnancy and postpartum, compared with PHIV on EFV.

**Conclusions:**

We observed high levels of obesity, elevated BP, and metabolic syndrome in the perinatal period but few differences by HIV status. Participants on DTG may be more likely to have elevated BP in pregnancy and postpartum. Monitoring of cardiometabolic health for pregnant persons on DTG is warranted.

Cardiometabolic complications in pregnancy, including obesity, dyslipidemia, and hypertensive disorders, adversely affect the health of pregnant persons and their developing fetuses and are associated with an increased risk of progression to cardiometabolic disease postpartum [1–5]. HIV influences cardiometabolic risk through chronic inflammation and sustained immune activation, which is reduced but not fully mitigated by antiretroviral therapy (ART) [6–8]. In South Africa, the ongoing HIV epidemic, coupled with rising rates of obesity in pregnancy [9], have led to increasing concerns about how cardiometabolic complications in pregnancy and postpartum may influence maternal, perinatal, and child health in the coming years.

In recent years, there has been growing concern about weight gain associated with integrase strand transfer inhibitors (INSTIs), with the largest increases in weight seen with dolutegravir (DTG) [10–15]. In 2019, the World Health Organization (WHO) recommended dolutegravir as first-line ART therapy for all pregnant persons with HIV (PHIV), and South Africa switched from efavirenz (EFV)-based ART to DTG-based ART later that year [16, 17]. Compared with other ART regimens, DTG has been associated with increased weight gain in pregnancy and postpartum, as well as hypertensive disorders in pregnancy [18–21]. DTG may affect cardiometabolic risk through weight gain, an increased risk of obesity, and insulin resistance [22, 23]. Despite these concerns, few data are available on the impact of DTG on the development of cardiometabolic risk factors in pregnancy and postpartum in South Africa.

To address this gap, we conducted a prospective cohort study among pregnant persons with and without HIV in Cape Town, South Africa. The study took place during the rollout of DTG in South Africa and allowed for comparisons of participants on EFV or DTG. We evaluated differences in cardiometabolic risk factors in pregnancy and postpartum including, obesity, hypertension, and lipid levels, by HIV status and ART regimen.

## METHODS

Data came from the Cardiometabolic Health in Pregnancy (CAMP) study, a prospective cohort study to investigate how HIV and ART regimen influence cardiometabolic risk in the perinatal period. The details of the CAMP study have been published [24]. Briefly, we enrolled pregnant persons living with and without HIV, who were ≥18 years of age, 24–28 weeks’ gestation, and presented for antenatal care (ANC) in Gugulethu in Cape Town, South Africa, between November 2019 and June 2022. Participants with and without HIV were enrolled in equal numbers, with no restriction on timing of ART initiation for PHIV. Participants completed 2 study visits at 24–28 weeks’ gestation (baseline) and a postpartum visit planned at 6 months postpartum. Due to the coronavirus disease 2019 (COVID-19) pandemic, the postpartum visit took place between 6 and 32 months postpartum (median [interquartile range {IQR}], 9.5 [6.8–12.2] months).

### Patient Consent

Participants provided written informed consent. Ethics approval for the CAMP study was provided by the University of Cape Town's Faculty of Health Sciences Human Research Ethics Committee (protocols 486/505).

Gugulethu is a peri-urban community in Cape Town with a population of ∼300 000, characterized by high levels of poverty and HIV among pregnant persons [25–27]. Access to antenatal care is nearly universal (>95%) and includes a recommended 6 prenatal visits [28]. ART for the prevention of mother-to-child HIV transmission is provided free of charge to PHIV as a part of routine antenatal care at public-sector clinics [26]. In 2013, all pregnant PHIV became eligible for same-day initiation of lifelong ART at entry into antenatal care, regardless of CD4 count or WHO clinical stage [29]. In June 2019, South Africa transitioned from initiating PHIV on EFV-based ART (tenofovir 300 mg + emtricitabine 200 mg + efavirenz 600 mg [TEE]) to DTG-based ART (tenofovir 300 mg + lamivudine 300 mg + dolutegravir 50 mg [TLD]) [17, 30]. Both regimens are provided free of charge as a fixed-dose combination pill taken once daily [30].

This aim of this analysis was to describe differences in cardiometabolic indicators in pregnancy and postpartum by HIV status and ART regimen (EFV vs DTG). Analyses in pregnancy include all enrolled participants (n = 400), and analyses postpartum were restricted to those with a postpartum visit (n = 296, 74%). One participant who seroconverted during follow-up was excluded from postpartum analyses (n = 295). Subgroup analyses comparing ART regimens were restricted to participants on EFV- or DTG-based ART (n = 193; 7 women on protease inhibitor [PI]–based ART were excluded from ART analyses). This approach is analogous to a “prevalent user” design [31], which is subject to selection bias [32], but more closely reflects clinical populations of PHIV on ART for varying lengths of time. To try to address bias due to the varying duration of ART, we conducted a sensitivity analysis restricted to PHIV initiating postconception EFV or DTG (n = 111/200).

### Data Collection

Gestational age at enrollment was determined primarily using ultrasound (360/400, 90%) in the first or second trimester. In some cases, last menstrual period and fundal height were used for women presenting later in pregnancy when ultrasound is less reliable [33, 34]. At each study visit, trained study staff measured anthropometry on participants in light-fitting clothing and no shoes on calibrated equipment using standardized procedures [35]. Prepregnancy BMI was estimated based on self-reported prepregnancy weight, which has been shown to be reasonably correlated with measured BMI [36], and study-measured height. Waist and hip circumference were measured at the postpartum study visit using an anthropometric tape. Blood pressure was evaluated at each study visit on seated participants using an automated blood pressure cuff that was appropriate for body size. Three measures, at least 30 minutes apart, were taken and were averaged for analyses. A lipid panel and plasma glucose assessments were completed using fasting blood samples by the National Health Laboratory Service in South Africa using standardized protocols.

### Exposures and Outcomes

The exposures of interest were HIV status and ART regimen during pregnancy. Information on HIV status and ART regimen was collected at baseline and confirmed via medical records. Outcomes of interest included several indicators of cardiometabolic risk: body mass index (BMI), obesity (BMI ≥30.0 kg/m^2^), blood pressure, and serum lipids in pregnancy and postpartum. We defined blood pressure outcomes in accordance with 2020 International Society of Hypertension Global Hypertension Practice guidelines [37]. Elevated blood pressure was considered ≥130 systolic mmHg, and/or ≥85 diastolic mmHg, with normal being <130 systolic mmHg and <85 diastolic mmHg. Grade 1 (≥140 and/or ≥90) and grade 2 (≥160 and/or ≥100) hypertension were considered secondary outcomes postpartum only due to few grade 1 or 2 hypertension outcomes (n = 12, 3%) in pregnancy [37]. We examined metabolic syndrome postpartum, which is a composite measure of cardiometabolic risk. Metabolic syndrome was defined according to Joint Interim Statement (JIS) criteria [38], with waist circumference defined based on previous work in South African women per JIS criteria [39], as ≥3 of the following: waist circumference >80 cm, triglycerides ≥1.7 mmol/L, high-density lipoprotein (HDL) cholesterol <1.3 mmol/L, elevated blood pressure (≥130 and/or ≥85), or fasting plasma glucose ≥5.6 mmol/L using laboratory values. Glucose metabolism and gestational weight gain outcomes were evaluated in separate analyses [24].

### Covariates

At baseline, information on clinical, behavioral, and HIV disease (if applicable) characteristics was collected by trained research assistants. Our group previously developed a composite socioeconomic status score, based on current employment, education, housing type, and access to household assets, that was used to categorize participants into tertiles of “highest, “moderate,” or “lowest” socioeconomic status (SES) [40]. Alcohol use was measured using the 3-item Alcohol Use Disorders Identification Test–Consumption (AUDIT-C; range 0–12). For women, an AUDIT-C score ≥3 indicates hazardous drinking in the previous 12 months [41]. Physical activity was evaluated as the number of times and intensity of physical activity in a week (light, moderate, or vigorous) using locally relevant examples (eg, walking, playing with children, or running). Household food security was assessed using adapted measures of the Household Food Insecurity Access Scale, Food and Nutrition Technical Assistance Project, and the Community Childhood Hunger Identification Project Index [42]. Tuberculosis status (defined as no previous tuberculosis, previous tuberculosis, or current tuberculosis) was defined based on medical records. Among people with HIV, ART adherence was assessed using a validated self-report measure [43] and timing of HIV diagnosis (during the current pregnancy or previously), and ART initiation (pre- or postconception) was assessed at baseline. Information on CD4 cell count (≤350, 351–≤500, >500 cells/mm^3^) and undetectable viral load (<50 copies/mL) was abstracted from medical records.

### Statistical Analyses

We graphically explored changes in the distribution of blood pressure (stratified by gestational age at enrollment) and lipids during pregnancy and postpartum by HIV- and ART status. Next, we estimated multivariable associations between HIV status or ART regimen and indicators of cardiometabolic risk using Poisson models with robust variance estimators for binary outcomes (eg, elevated blood pressure) [44] and linear regression for continuous outcomes (eg, continuous systolic or diastolic blood pressure). All models were adjusted for a minimally sufficient adjustment set of confounders identified using directed acyclic graphs (DAG) (see Supplementary Figure 1 for a sample DAG) [45]. Due to multicollinearity issues between age and parity, all multivariable models were adjusted for age and all other potential confounders. The cohort was powered to detect differences in blood pressure outcomes by HIV status. Assuming an 8% prevalence of elevated blood pressure [46] with a sample size of 400, we had 81% power to detect a 10% absolute increase in risk (relative risk, 2.25) in elevated blood pressure among participants with HIV. To address potential bias due to the varying timing of the postpartum visit, we conducted sensitivity analyses restricted to participants with a postpartum visit within 18 months (n = 270/295), when most cardiometabolic changes from pregnancy (eg, weight, blood pressure) have stabilized [47, 48] and compared sociodemographic characteristics of all participants with those with a postpartum visit. Except for viral load (56%) and CD4 count (21%), which were available as part of routine care from medical records, missing data were limited (≤3%), and therefore all analyses were complete case. Statistical analyses were conducted in Stata, version 15 (StataCorp, College Station, TX, USA).

## RESULTS

We enrolled 400 participants (n = 200 without HIV, n = 200 PHIV) during pregnancy (median [IQR] gestational age, 26 [24–27] weeks). PHIV had slightly higher parity (median, 3 vs 2) and were more likely to be in the “lowest” SES category (39% vs 26%) compared with participants without HIV (Table 1). There was little difference in hazardous alcohol use, physical activity, or perceived food insecurity by HIV status. Among PHIV, 88 (44%) initiated ART preconception (n = 71 EFV, n = 11 DTG, n = 6 PI regimen) and 112 (56%) initiated ART postconception (n = 36 EFV, n = 75 DTG, n = 1 PI regimen). Among all PHIV, EFV users were on ART longer before study enrollment (mean [SD], 210.3 [199.7] weeks) compared with DTG users (mean [SD], 11.8 [39.5] weeks). Among PHIV initiating ART postconception, participants were on treatment for an average (SD) of 6.7 (5.1) weeks at study enrollment (EFV: mean [SD], 10.4 [4.8] weeks; DTG: mean [SD], 5.0 [4.3] weeks). About a third of PHIV with measures available had a CD4 count ≤350 cells/mm^3^ or a detectable viral load (≥50 copies/mL) at enrollment.

**Table 1.** Characteristics at 24–28 Weeks’ Gestation Among 400 Pregnant Persons in Cape Town, South Africa, Overall and by HIV Status

	Without HIV (n = 200)	With HIV (n = 200)	Total (n = 400)
	Median (IQR)		
Age, y	27 (24–31)	31 (27–36)	30 (25–34)
Gestational age, wk	26 (24–27)	26 (24–27)	26 (24–27)
Parity	2 (1–3)	3 (2–4)	3 (2–3)
Prepregnancy BMI, kg/m^2a^	31.1 (26.2–35.9)	29.2 (25.3–34.2)	30.1 (25.8–35.0)
Blood pressure, mm/Hg			
Systolic	113.8 (105.5–121.3)	111.5 (103.6–119.8)	112.3 (104.7–120.5)
Diastolic	67.0 (62.5–71.5)	66.8 (62.6–72.9)	67.0 (62.5–72.3)
Fasting lipids, mmol/L			
Total cholesterol	4.8 (4.2–5.5)	4.5 (3.9–5.1)	4.6 (4.0–5.3)
LDL cholesterol	2.4 (1.8–2.9)	2.1 (1.6–2.6)	2.3 (1.7–2.8)
HDL cholesterol	1.7 (1.4–1.9)	1.6 (1.4–1.9)	1.6 (1.4–1.9)
Triglycerides	1.5 (1.2–1.8)	1.6 (1.3–2.0)	1.5 (1.3–1.9)
	No. (%)		
Prepregnancy BMI category, kg/m^2a^			
Underweight (<18.5)	0 (0.0)	0 (0.0)	0 (0.0)
Normal (18.5–< 25.0)	37 (18.6)	42 (21.0)	79 (19.8)
Overweight (25.0–< 30.0)	51 (25.6)	62 (31.0)	113 (28.3)
Obese (≥30.0)	111 (55.8)	96 (48.0)	207 (51.9)
SES category			
Lowest	51 (25.5)	78 (39.0)	129 (32.3)
Moderate	58 (29.0)	51 (25.5)	109 (27.2)
Highest	91 (45.5)	71 (35.5)	162 (40.5)
Marital status			
Not married/cohabitating	114 (57.0)	112 (56.0)	226 (56.5)
Married/cohabitating	86 (43.0)	88 (44.0)	174 (43.5)
Primigravida			
No	149 (18.5)	177 (88.5)	326 (81.5)
Yes	51 (25.5)	23 (11.5)	74 (18.5)
Alcohol use^b^			
Below threshold	188 (94.0)	188 (94.0)	376 (94.0)
Hazardous drinking	12 (6.0)	12 (6.0)	24 (6.0)
Food security^c^			
None	172 (86.0)	161 (80.5)	333 (83.3)
Perceived food insecurity	28 (14.0)	39 (19.5)	67 (16.7)
Physical activity			
None	104 (52.0)	77 (38.5)	181 (45.3)
1–2 times/wk	52 (26.0)	48 (24.0)	100 (25.0)
3–4 times/wk	35 (17.5)	58 (29.0)	93 (23.2)
>4 times/wk	9 (4.5)	17 (8.5)	26 (6.5)
Physical activity intensity (among women who engage in physical activity n = 219)			
Light	81 (84.4)	113 (91.9)	194 (88.6)
Moderate	10 (10.4)	9 (7.3)	19 (8.7)
Vigorous	5 (5.2)	1 (0.8)	6 (2.7)
Tuberculosis			
No tuberculosis	190 (95.0)	171 (85.5)	361 (90.3)
Previous tuberculosis	10 (5.0)	28 (14.0)	38 (9.5)
Current tuberculosis	0 (0.0)	1 (0.5)	1 (0.2)
Maternal HIV Characteristics (n = 200)			
	Preconception ART n = 88	Postconception ART n = 112	With HIV n = 200
HIV diagnosis	No. (%)		
Before this pregnancy, but during another pregnancy	47 (53.4)	19 (17.0)	66 (33.0)
Before this pregnancy, but not during another pregnancy	41 (46.6)	27 (24.1)	68 (34.0)
During this pregnancy	0 (0.0)	65 (58.0)	65 (32.5)
Perinatally infected	0 (0.0)	1 (0.9)	1 (0.5)
ART regimen			
Efavirenz based	71 (80.6)	36 (32.1)	107 (53.5)
Dolutegravir based	11 (12.5)	75 (67.0)	86 (43.0)
Other	6 (6.8)	1 (0.9)	7 (3.5)
Viral load			
Undetectable (<50 copies/mL)	60 (84.5)	2 (11.1)	62 (69.7)
Detectable (≥50 copies/mL)	11 (15.5)	16 (88.9)	27 (30.3)
CD4 count, cells/mm^3^			
≤350	9 (12.0)	38 (45.2)	47 (29.6)
351–≤500	23 (30.7)	19 (22.6)	42 (26.4)
>500	43 (57.3)	27 (32.1)	70 (44.0)

Abbreviations: ART, antiretroviral therapy; AUDIT-C, Alcohol Use Disorders Identification Test–Consumption; BMI, body mass index; GA, gestational age; SES, socioeconomic status; WHO, World Health Organization.

^a^Based on WHO categories and self-reported prepregnancy weight.

^b^Based on the AUDIT-C (range, 0–12), a score of ≥3 indicates hazardous drinking.

^c^Household food security was assessed using adapted measures of the Household Food Insecurity Access Scale, Food and Nutrition Technical Assistance Project, and the Community Childhood Hunger Identification Project Index. Missing data: prepregnancy BMI n = 2 (0.5%); family history of diabetes n = 12 (3.0%); CD4 cell count n = 41 (21%); viral load n = 111 (56%).

### BMI and Obesity

An estimated 52% of participants entered pregnancy with obesity, which increased to 57% with obesity by a median of 10 months postpartum. In multivariable analyses, HIV was associated with a slightly lower BMI in both prepregnancy but not postpartum. However, mean prepregnancy and postpartum BMI levels were in the obese range regardless of HIV status (Tables 2 and 3). PHIV were less likely to be obese prepregnancy, and this risk was attenuated postpartum (Table 3).

**Table 2.** Cardiometabolic Health Indicators at 24–28 Weeks’ Gestation, by HIV Status and ART Regimen

	Without HIV (n = 200)	With HIV (n = 200)	Full Cohort (n = 400)	Efavirenz (n = 107)	Dolutegravir (n = 86)	Total (n = 193)
	No. (%)		Risk Ratio (95% CI)	No. (%)		Risk Ratio (95% CI)
Prepregnancy obesity	111 (55.8)	96 (48.0)	0.82 (0.68, 0.99)^a,b^	53 (49.5)	39 (45.4)	0.96 (0.70, 1.32)^1^
Elevated blood pressure	19 (9.6)	18 (9.0)	1.11 (0.58, 2.10)^c^	2 (1.9)	15 (17.4)	–^d^
	Mean (SD)		Mean Difference (95% CI)	Mean (SD)		Mean Difference (95% CI)
Prepregnancy BMI, kg/m^2^	31.2 (6.3)	30.1 (6.4)	−2.03 (−3.33, −0.71)^a^	30.0 (6.1)	30.2 (6.7)	0.37 (−1.48, 2.22)^a^
Blood pressure, mm/Hg						
Systolic	113.9 (11.0)	112.7 (12.4)	0.18 (−2.18, 2.54)^c^	109.8 (9.6)	115.7 (14.8)	5.01 (1.68, 8.35)^c^
Diastolic	67.8 (7.7)	68.0 (8.1)	0.22 (−1.42, 1.87)^c^	66.6 (6.4)	69.4 (9.7)	2.18 (−0.06, 4.43)^c^
Lipids, mmol/L						
Total cholesterol	4.8 (0.9)	4.6 (0.9)	−0.26 (−0.46, −0.07)^e^	4.6 (0.9)	4.5 (0.9)	−0.01 (−0.25, 0.28)^e^
LDL cholesterol	2.4 (0.8)	2.2 (0.8)	−0.23 (−0.41, −0.06)^e^	2.1 (0.8)	2.3 (0.8)	0.27 (0.04, 0.50)^e^
HDL cholesterol	1.7 (0.3)	1.6 (0.4)	−0.06 (−0.14, 0.01)^e^	1.7 (0.4)	1.5 (0.3)	−0.20 (−0.30, −0.10)^e^
Triglycerides	1.6 (0.5)	1.7 (0.6)	0.06 (−0.06, 0.17)^e^	1.7 (0.6)	1.6 (0.4)	−0.11 (−0.26, 0.03)^e^

Obesity defined as BMI ≥30 kg/m^2^; elevated blood pressure defined as systolic ≥130 and/or diastolic ≥85 mmHg.

Abbreviations: ART, antiretroviral therapy; BMI, body mass index; HDL, high-density lipoprotein; LDL, low-density lipoprotein.

^a^Adjusted for food insecurity status, tuberculosis status, physical activity frequency, age, socioeconomic status.

^b^Model adjusted for parity instead of age, due to model convergence issues.

^c^Adjusted for prepregnancy BMI, food insecurity status, tuberculosis status, physical activity frequency, socioeconomic status, alcohol use, and age.

^d^Model cannot be estimated. Missing data: prepregnancy BMI n = 1, systolic and diastolic blood pressure n = 1, all lipids n = 1, except LDL cholesterol n = 2.

^e^Adjusted for prepregnancy BMI, food insecurity status, tuberculosis status, physical activity frequency, age, socioeconomic status.

**Table 3.** Cardiometabolic Health Indicators at 6–32 Months Postpartum, by HIV Status and ART Regimen

	Without HIV (n = 144)	With HIV (n = 151)	Full Cohort (n = 295)	Efavirenz (n = 74)	Dolutegravir (n = 71)	On ART (n = 145)
	No. (%)		Risk Ratio (95% CI)	No. (%)		Risk Ratio (95% CI)
Obesity	87 (62.8)	76 (52.1)	0.94 (0.79, 1.07)^a^	41 (56.9)	31 (45.6)	0.85 (0.65, 1.12)^a^
Metabolic syndrome	39 (27.9)	30 (20.6)	0.76 (0.49, 1.16)^a^	12 (16.7)	17 (25.0)	1.21 (0.64, 2.27)^a^
Metabolic syndrome defined as ≥3 of the following:						
Waist circumference >80 cm	129 (94.9)	135 (92.5)	–	68 (94.4)	62 (91.2)	–
Triglycerides ≥1.7mmol/L	3 (2.2)	5 (3.5)	–	3 (4.2)	2 (3.0)	–
HDL cholesterol <1.3 mmol/L	86 (63.7)	86 (59.7)	–	41 (56.9)	43 (63.2)	–
Elevated blood pressure (systolic ≥130 and/or diastolic ≥85 mmHg)	44 (32.1)	45 (30.8)	–	14 (19.4)	27 (39.7)	–
Fasting plasma glucose (≥5.6 mmol/L)	7 (5.2)	7 (4.9)	–	6 (8.3)	1 (1.5)	–
Elevated blood pressure	44 (32.1)	45 (30.8)	0.88 (0.62, 1.24)^b^	14 (19.4)	27 (39.7)	1.83 (1.05, 3.22)^b^
Grade 1 hypertension	17 (12.4)	19 (13.0)	0.72 (0.38, 1.38)^b^	8 (11.1)	11 (16.2)	1.23 (0.50, 2.99)^b^
	Mean (SD)		Mean Difference (95% CI)	Mean (SD)		Mean Difference (95% CI)
BMI, kg/m^2^	33.8 (7.2)	31.3 (7.7)	−0.81 (−1.68, 0.07)^a^	31.7 (7.4)	30.5 (7.3)	−0.95 (−2.02, 0.11)^a^
Weight, kg	86.6 (19.0)	80.1 (20.7)	−2.49 (−5.33, 0.36)^a^	80.6 (19.1)	78.4 (20.5)	−1.57 (−5.36, 2.23)^a^
Blood pressure, mm/Hg						
Systolic	122.8 (13.4)	121.8 (13.7)	−0.98 (−4.27, 2.30)^b^	119.3 (12.6)	124.1 (14.8)	4.25 (−0.19, 8.70)^b^
Diastolic	74.7 (10.5)	75.5 (9.1)	−0.40 (−2.82, 2.02)^b^	74.2 (7.9)	76.6 (10.2)	1.87 (−1.16, 4.91)^b^
Lipids, mmol/L						
Total cholesterol	4.0 (0.8)	3.8 (0.8)	−0.29 (−0.50, −0.08)^a^	3.8 (0.9)	3.6 (0.7)	−0.18 (−0.45, 0.09)^a^
LDL cholesterol	2.3 (0.7)	2.1 (0.7)	−0.27 (−0.45, −0.09)^a^	2.1 (0.8)	2.0 (0.6)	−0.06 (−0.29, 0.17)^a^
HDL cholesterol	1.3 (0.4)	1.3 (0.4)	−0.05 (−0.15, 0.05)^a^	1.3 (0.4)	1.3 (0.4)	−0.04 (−0.17, 0.09)^a^
Triglycerides	0.8 (0.3)	0.9 (0.5)	0.06 (−0.05, 0.16)^a^	0.9 (0.4)	0.8 (0.5)	−0.09 (−0.25, 0.07)^a^

Obesity defined as BMI ≥30 kg/m^2^; elevated blood pressure defined as systolic ≥130 and/or diastolic ≥85 mmHg; grade 1 hypertension defined as ≥140 and/or ≥90 mmHg.

Abbreviations: ART, antiretroviral therapy; BMI, body mass index; HDL, high-density lipoprotein; LDL, low-density lipoprotein.

^a^Adjusted for prepregnancy BMI, food insecurity status, tuberculosis status, physical activity frequency, age, socioeconomic status.

^b^Adjusted for prepregnancy BMI, food insecurity status, tuberculosis status, physical activity frequency, socioeconomic status, alcohol use, age. Missing data: n = 12 for BMI, weight, systolic and diastolic blood pressure, n = 9 for metabolic syndrome, and n = 16 for lipids.

Among PHIV, there were no differences in prepregnancy or postpartum BMI or obesity levels by ART regimen (Tables 2 and 3). Participants on both DTG and EFV had lower mean weight and BMI prepregnancy and postpartum than participants without HIV. Results were similar when restricted to PHIV initiating postconception ART only (Supplementary Tables 1 and 2).

### Blood Pressure

**Figure 1.**
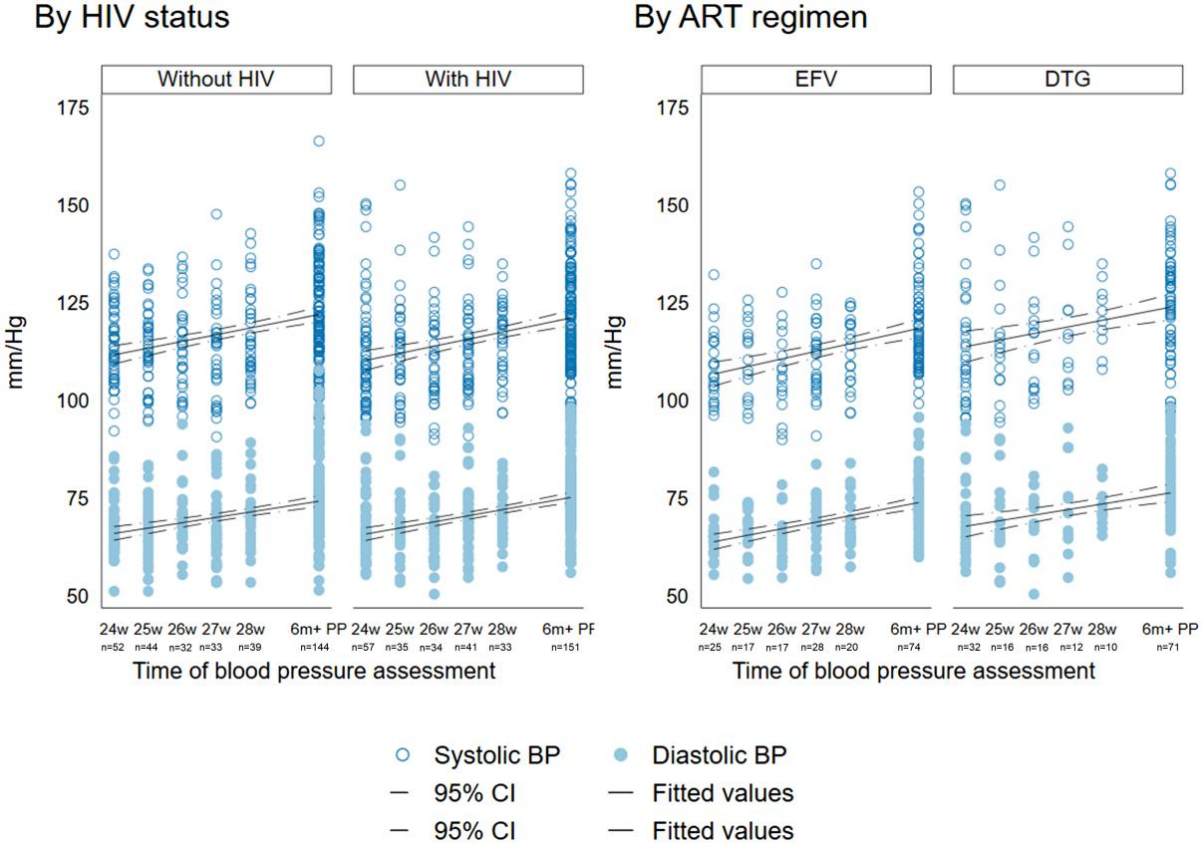
Systolic and diastolic blood pressure at gestational age of enrollment in pregnancy and 6–32 months postpartum, by HIV status and ART regimen. Gestational age measured in weeks. Abbreviations: ART, antiretroviral therapy; BP, blood pressure.

At 24–28 weeks’ gestation, 9% of participants had elevated blood pressure. This increased to 31% postpartum, including 11% with grade 1 hypertension and 1% with grade 2 hypertension. In multivariable analyses, there was no evidence that HIV status was associated with differences in systolic or diastolic blood pressure, elevated blood pressure in pregnancy or postpartum, or grade 1 hypertension postpartum (Tables 2 and 3). Blood pressure levels were lower in pregnancy than postpartum and tended to rise slightly with increasing gestational age, regardless of HIV status (Figure 1).

At 24–28 weeks’ gestation, participants on DTG had higher systolic blood pressure, and a higher proportion had elevated blood pressure, compared with participants on EFV (Table 2). Higher levels of elevated blood pressure among participants on DTG was also observed at the postpartum visit, with a trend toward higher systolic blood pressure after adjustment for confounders (Table 3). Associations between DTG, vs EFV, and blood pressure outcomes were in the same direction, but attenuated, in a sensitivity analysis restricted to PHIV on postconception DTG or EFV (Supplementary Tables 1 and 2).

### Lipids

**Figure 2.**
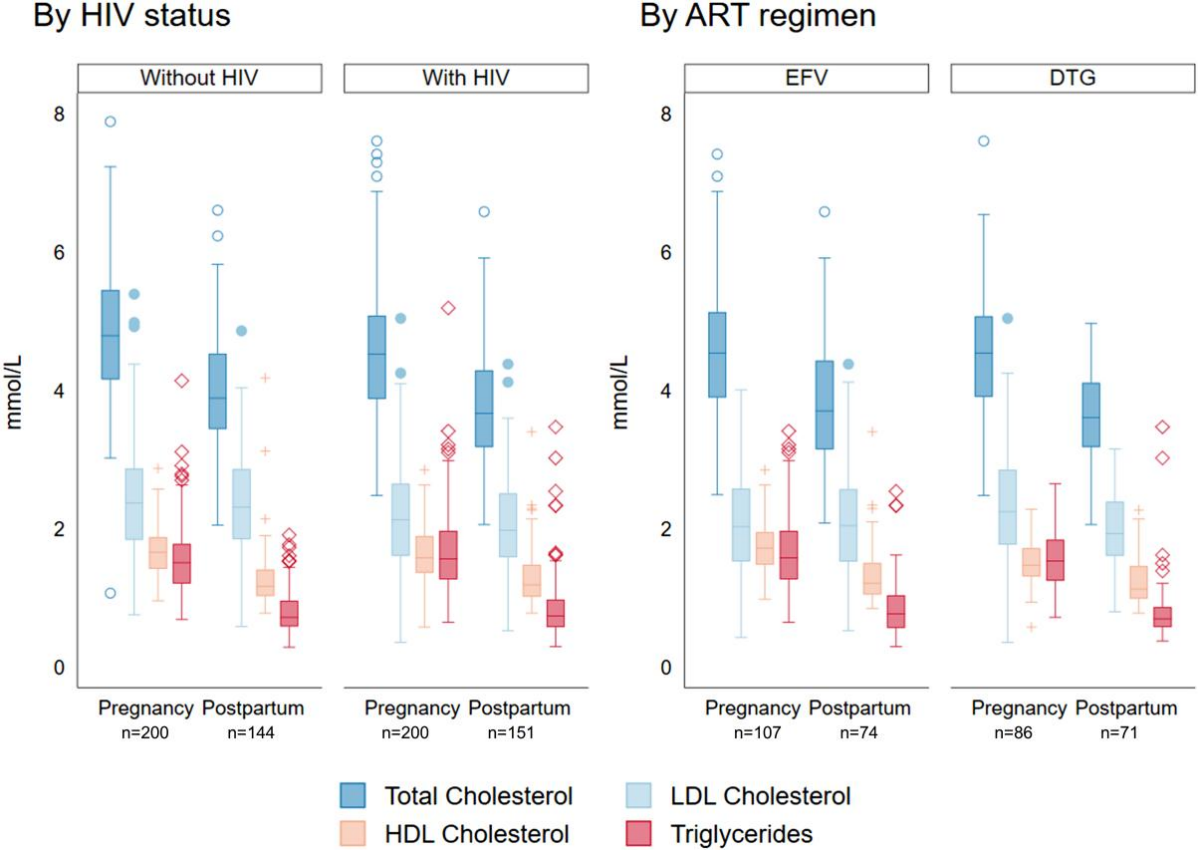
Lipid levels at 24–28 weeks’ gestation in pregnancy and 6–26 months postpartum by HIV status and ART regimen. Abbreviations: ART, antiretroviral therapy; DTG, dolutegravir; EFV, efavirenz; HDL, high-density lipoprotein; LDL, low-density lipoprotein.

Compared with participants without HIV, PHIV had slightly lower total and LDL cholesterol in pregnancy and postpartum (Tables 2 and 3). Overall, lipid levels tended to be lower in the postpartum period compared with in pregnancy, regardless of HIV status (Figure 2). Compared with participants on EFV, those on DTG had slightly lower levels of HDL cholesterol and higher levels of LDL cholesterol in pregnancy. Postpartum, there were no differences in lipid levels by ART regimen (Table 3).

### Metabolic Syndrome

By the postpartum visit, 24% of participants met the definition of metabolic syndrome. The most common individual risk factors contributing to metabolic syndrome (defined as ≥3 of the following risk factors) were waist circumference >80 cm (94%), HDL cholesterol <1.3 mmol/L (62%), and elevated blood pressure (systolic ≥130 and/or diastolic ≥85 mmHg; 31%). In multivariable analyses, there was no evidence that the risk of metabolic syndrome differed by HIV status or ART regimen in pregnancy (Table 3). For all postpartum cardiometabolic outcomes, results did not meaningfully change when analyses were restricted to participants with a visit within 18 months postpartum (Supplementary Table 3).

## DISCUSSION

In a prospective cohort of pregnant persons living with and without HIV in South Africa, we observed high levels of cardiometabolic risk factors in pregnancy and postpartum, including obesity, elevated blood pressure, and metabolic syndrome. Despite the high overall levels of cardiometabolic risk factors, there was little evidence that PHIV were more likely to experience adverse cardiometabolic outcomes compared with participants without HIV. Conversely, we observed higher systolic blood pressure and higher levels of elevated blood pressure among participants on DTG in pregnancy and postpartum, despite similar BMIs at baseline to those on EFV and adjustment for other confounders. When restricted to postconception ART initiators, associations of DTG with elevated blood pressure were in the same direction but attenuated.

A growing body of evidence in pregnant and nonpregnant populations has linked INSTIs, and specifically DTG, to weight gain [10–15, 49]. In this cohort, participants initiating DTG and EFV had similar BMIs prepregnancy, and there was little difference in BMI or the risk of obesity postpartum by ART regimen. These findings differ from observational studies in Sub-Saharan Africa and the IMPAACT 2010 trial, which found that PHIV on DTG weighed more at 12–18 months postpartum than those on EFV-based ART [18, 19, 50], but they align with evidence from a nonpregnant South African cohort showing modest weight gain (mean, 1.8 kg over 12 months) after switching from EFV to DTG [51]. In this study, participants on both EFV and DTG had lower average postpartum weights and BMIs than participants without HIV, similar to findings from Botswana [18].

DTG-associated weight gain may influence hypertension risk [52]. INSTIs have been associated with an increased risk of hypertensive disorders in pregnancy [21] and elevated blood pressure and hypertension in several nonpregnant cohorts [51, 53–55]. In this cohort, participants on DTG had higher systolic blood pressure in pregnancy and a higher risk of elevated blood pressure in pregnancy and postpartum, after adjustment for BMI, age, and other confounders. These differences persisted, but were attenuated, when analyses were restricted to participants initiating postconception EFV or DTG. A potential increased risk of hypertension with DTG may be mediated by increases in body weight, especially central adiposity [53, 56]. However, in this study, PHIV initiating DTG did not weigh more than PHIV initiating EFV in pregnancy or postpartum, although we lacked detailed data on body composition. Further, analyses of changes in body composition and hypertension risk among people on DTG are needed to understand potential mechanisms of action [57].

Our finding that the risk of elevated blood pressure in pregnancy did not differ by HIV status largely aligns with previous literature on HIV and hypertensive disorders in pregnancy [21, 58–60], including a recent population-based cohort study form the Western Cape province in South Africa [61]. While these findings are reassuring, the fact that a third of the cohort had elevated blood pressure or higher postpartum is concerning. Outside of HIV/AIDS, heart disease and stroke are the leading causes of death in South Africa [62]. Our findings suggest that postpartum hypertension screening could help to identify persons with elevated blood pressure for behavior change counseling and ongoing monitoring.

Obesity before pregnancy is increasing in low- and middle-income countries, including South Africa [9], and has the potential to adversely affect cardiometabolic health for pregnant persons and their children. In this cohort, over half of participants entered pregnancy with obesity, and by the postpartum period nearly 60% were living with obesity, with no evidence of differences by HIV status. EFV has been associated with higher trunk fat and an increased risk of dyslipidemia [63]. In this cohort, participants on EFV also had slightly higher lipid levels than DTG users. In addition, the majority of PHIV (81%) on preconception ART were on EFV. This longer duration of EFV exposure, along with the high rates of obesity, may help to explain the high prevalence of metabolic syndrome observed in this cohort.

Reducing obesity, metabolic syndrome, and hypertension in the perinatal period is critical to reduce the risk of cardiometabolic disease in later life for people living with and without HIV. Lifestyle interventions targeting changes in diet or physical activity are effective in reducing excessive weight gain in pregnancy [64] and in reducing diabetes and cardiovascular risk in nonpregnant populations [65–68]. To date, few interventions have been developed, adapted, or tested in low- and middle-income country settings or in the context of high HIV burden. Additional work on intervention development and evaluation to address cardiometabolic risk in the perinatal period for women with HIV (such as potential ART-associated weight gain) is urgently needed.

Our study has several strengths and limitations. The strengths include the use of study-collected blood pressure and anthropometry measures and the ability to follow participants into the postpartum period. Our study also provides some of the first evidence on the cardiometabolic impact of DTG- vs EFV-based ART in pregnancy and postpartum. Limitations include the heterogenous duration of ART among all PHIV, which could lead to selection bias if length of ART use is associated with surviving long enough to take part in the study [32]. However, mortality is not likely to lead to appreciable selection bias in this cohort of young people healthy enough to get pregnant. To try to reduce bias due to varying duration of ART, we restricted the sensitivity analysis to postconception DTG and EFV users. This approach reduces potential selection bias, but it is limited by the short duration (∼7 weeks) of ART use among postconception ART initiators. In the ADVANCE trial, differences in weight between EFV and DTG were evident as early as 4 weeks postinitiation [12]. Further, findings of initiating DTG, vs EFV, on cardiometabolic indicators in pregnancy largely align with findings postpartum, when ART duration would have been longer. We note the small sample size, limited information on viral load suppression, inability to account for possible changes in ART regimen over time, and low postpartum follow-up (74%) due to the COVID-19 pandemic as limitations. However, there were no important differences in sociodemographic characteristics between the full cohort and those with a postpartum visit (Supplementary Table 4), and associations with cardiometabolic indicators were virtually unchanged when restricted to participants with a visit within 18 months postpartum. Our study took place in part during the COVID-19 pandemic, but we did not have data on COVID-19 exposure or treatment. SARS-CoV-2 seroprevalence among pregnant people in South Africa is estimated at >60% and is typically asymptomatic [69–71]. Finally, we did not have data on cardiovascular medication during pregnancy and were unable to distinguish between chronic and pregnancy-induced hypertension. Chronic hypertension affects an estimated 0.9%–1.5% of pregnancies [72], suggesting that our estimates primarily reflect pregnancy-induced increases in blood pressure.

## CONCLUSIONS

In this prospective cohort of pregnant persons living with and without HIV in South Africa, we observed high levels of obesity, elevated blood pressure, and metabolic syndrome in the perinatal period, but few differences by HIV status. Participants on DTG had a higher likelihood of elevated blood pressure in midpregnancy, and this trend persisted into the postpartum period. Given concerns about DTG, weight gain, and elevated blood pressure in other cohorts, the findings from our study should be confirmed in larger cohorts. If confirmed, hypertension screening among people with HIV initiating or switching to DTG during pregnancy and postpartum may be warranted. These findings highlight the need for interventions to address the growing issue of obesity and its sequelae in pregnancy in low- and middle-income countries for persons living with and without HIV.

## Supplementary Material

ofae093_Supplementary_Data

## Supplementary Data

Supplementary materials are available at *Open Forum Infectious Diseases* online. Consisting of data provided by the authors to benefit the reader, the posted materials are not copyedited and are the sole responsibility of the authors, so questions or comments should be addressed to the corresponding author.
