# Supplementary material for: Cardiometabolic Health in Pregnancy and Postpartum: Findings From a Prospective Cohort Study in South Africa
Source: Open Forum Infect Dis. 2024 Feb 22;11(3):ofae093. doi: 10.1093/ofid/ofae093 (PMC10932940; doi:10.1093/ofid/ofae093)
Supplement: ofae093_Supplementary_Data [file ofae093_supplementary_data.docx]

**Supplemental Tables and Figures**

**Figure S1. Sample directed acyclic graph (DAG) for the relationship between HIV status and elevated blood pressure.**


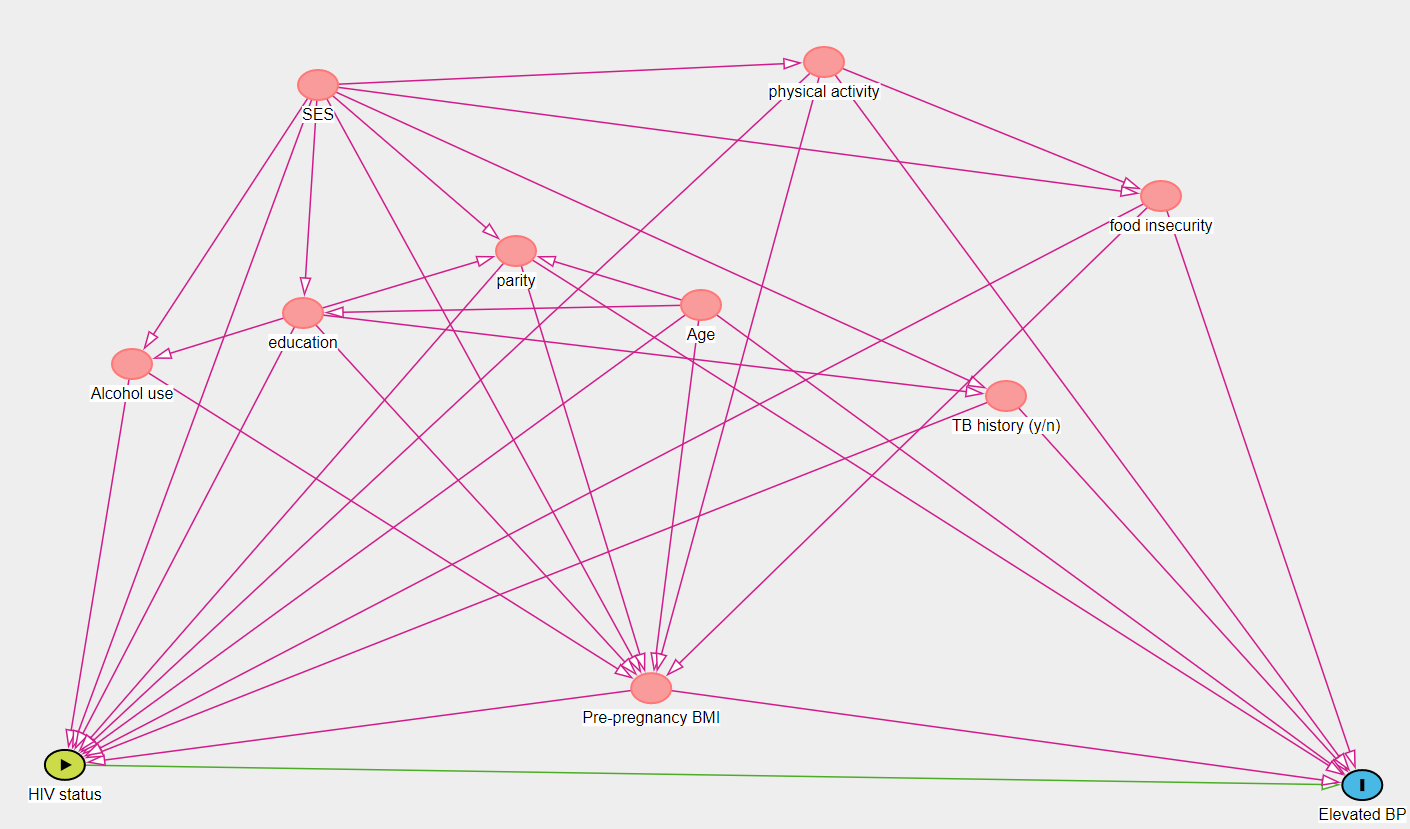


| **Table S1. Cardiometabolic health indicators at 24-28 weeks gestation, by ART regimen among post-conception ART initiators.** | | | |
| --- | --- | --- | --- |
|  | **Efavirenz  (n=36)** | **Dolutegravir  (n=75)** | **Post-conception ART  (N=111)** |
|  | **N (%)** | | **Risk Ratio  (95% CI)** |
| **Pre-pregnancy obesity** | 16 (44.4) | 35 (46.7) | 1.02 (0.66, 1.57)^1*^ |
| **Elevated blood pressure** | 0 (0.0) | 13 (17.3) | --** |
|  | **Mean (SD)** | | **Mean difference (95% CI)** |
| **Pre-pregnancy BMI (kg/m^2^)** | 28.8 (5.8) | 30.5 (6.7) | 1.04 (-1.50, 3.57)^1^ |
| **Blood pressure, mm/Hg** |  |  |  |
| Systolic | 111.5 (9.3) | 115.7 (15.4) | 2.70 (-2.55, 7.94)^2^ |
| Diastolic | 67.0 (6.0) | 69.4 (9.8) | 1.29 (-2.18, 4.76)^2^ |
| **Lipids, mmol/l** |  |  |  |
| Total cholesterol | 4.7 (0.9) | 4.5 (1.0) | -0.14 (-0.55, 0.26)3 |
| LDL cholesterol | 2.2 (0.8) | 2.3 (0.9) | 0.11 (-0.24, 0.46)^3^ |
| HDL cholesterol | 1.7 (0.3) | 1.5 (0.3) | -0.16 (-0.29, -0.03)^3^ |
| Triglycerides | 1.7 (0.5) | 1.6 (0.4) | -0.22 (-0.41, -0.04)3 |
| ART: antiretroviral therapy; BMI: body mass index; LDL: low density lipoprotein; HDL: high density lipoprotein. Obesity defined as BMI≥30kg/m^2^; Elevated blood pressure defined as systolic ≥130 and/or diastolic ≥85 mmHg.  ^1^ Adjusted for food insecurity status, tuberculosis status, physical activity frequency, age, socioeconomic status. ^2^ Adjusted for: pre-pregnancy BMI, food insecurity status, tuberculosis status, physical activity frequency, socioeconomic status, alcohol use, and age. ^3^ Adjusted for: pre-pregnancy BMI, food insecurity status, tuberculosis status, physical activity frequency, age, socioeconomic status. *model estimated with parity instead of age due to model convergence issues. **model cannot be estimated. Missing data: Pre-pregnancy BMI n=1, systolic and diastolic blood pressure n=1, all lipids n=1, except LDL cholesterol n=2. | | | |

| **Table S2. Cardiometabolic health indicators at 6-32 months postpartum,  by ART regimen among post-conception ART initiators.** | | | |
| --- | --- | --- | --- |
|  | **Efavirenz  (n=22)** | **Dolutegravir  (n=61)** | **Post-conception ART  (N=83)** |
|  | **N (%)** | | **Risk Ratio  (95% CI)** |
| **Obesity** | 11 (52.4) | 29 (49.2) | 0.70 (0.46, 1.06)^1^ |
| **Metabolic syndrome** | 2 (9.5) | 17 (28.8) | 1.90 (0.48, 7.57)1 |
| **Elevated blood pressure** | 5 (23.8) | 26 (44.1) | 1.47 (0.65, 3.33)2 |
| **Grade 1 hypertension** | 4 (19.1) | 10 (17.0) | 0.52 (0.15,1.78)2 |
|  | **Mean (SD)** | | **Mean difference (95% CI)** |
| **BMI (kg/m^2^)** | 29.7 (6.2) | 31.1 (7.3) | -0.27 (-1.84, 1.29)1 |
| **Weight (kg)** | 76.1 (16.6) | 79.9 (20.8) | -0.64 (-6.84, 5.55)1 |
| **Blood pressure, mm/Hg** |  |  |  |
| Systolic | 123.0 (13.3) | 125.8 (14.1) | 0.28 (-6.70, 7.26)^2^ |
| Diastolic | 75.1 (8.8) | 77.5 (9.6) | 0.98 (-3.97, 5.93)^2^ |
| **Lipids, mmol/l** |  |  |  |
| Total cholesterol | 3.9 (0.7) | 3.7 (0.7) | -0.28 (-0.63, 0.07)1 |
| LDL cholesterol | 2.1 (0.5) | 2.0 (0.6) | -0.18 (-0.46, 0.11)1 |
| HDL cholesterol | 1.4 (0.4) | 1.3 (0.4) | -0.06 (-0.25, 0.13)1 |
| Triglycerides | 0.8 (0.3) | 0.8 (0.5) | -0.05 (-0.29, 0.20)1 |
| ART: antiretroviral therapy; BMI: body mass index; LDL: low density lipoprotein; HDL: high density lipoprotein. Obesity defined as BMI≥30kg/m^2^; Elevated blood pressure defined as systolic ≥130 and/or diastolic ≥85 mmHg; Grade 1 hypertension defined as ≥140 and/or ≥90 mmHg. ^1^ Adjusted for: pre-pregnancy BMI, food insecurity status, tuberculosis status, physical activity frequency, age, socioeconomic status. ^2^ Adjusted for: pre-pregnancy BMI, food insecurity status, tuberculosis status, physical activity frequency, socioeconomic status, alcohol use, and age. Missing data: n=3 for BMI, weight, systolic and diastolic blood pressure and n=4 for lipids. | | | |

| **Table S3. Cardiometabolic health indicators at 6-18 months postpartum,  by HIV status and ART regimen.** | | | | | |  |  |  |  |  |
| --- | --- | --- | --- | --- | --- | --- | --- | --- | --- | --- |
|  | **Without HIV (n=129)** | | **With HIV  (n=141)** | | **Full Cohort (N=270)** | |  | **Efavirenz  (n=67)** | **Dolutegravir  (n=70)** | **On ART  (N=137)** |
|  | **N (%)** | | | | **Risk Ratio  (95% CI)** | |  | **N (%)** | | **Risk Ratio  (95% CI)** |
| **Obesity** | 79 (62.4) | | 72 (51.8) | | 0.97 (0.80, 1.17)1 | |  | 38 (56.7) | 31 (45.6) | 0.81 (0.61, 1.09)^1^ |
| **Metabolic syndrome** | 34 (26.8) | | 27 (19.4) | | 0.83 (0.52, 1.33)1 | |  | 9 (13.4) | 17 (25.0) | 1.29(0.63, 2.66)1 |
| **Elevated blood pressure** | 38 (30.2) | | 43 (30.9) | | 0.95 (0.65, 1.39)^2^ | |  | 13 (19.4) | 27 (39.7) | 1.84 (1.04, 3.26)2 |
| **Grade 1 hypertension** | 15 (11.9) | | 18 (13.0) | | 0.68 (0.34, 1.35)^2^ | |  | 7 (10.5) | 11 (16.2) | 1.50 (0.63, 3.59)2 |
|  | **Mean (SD)** | | | | **Mean difference (95% CI)** | |  | **Mean (SD)** | | **Mean difference  (95% CI)** |
| **BMI (kg/m^2^)** | 33.7 (7.3) | | 31.0 (7.2) | | -0.82 (-1.72, 0.08)1 | |  | 31.2 (6.8) | 30.5 (7.3) | -0.85 (-1.88, 0.17)1 |
| **Weight (kg)** | 86.4 (19.2) | | 79.4 (19.7) | | -2.42 (-5.39, 0.55)1 | |  | 79.4 (17.9) | 78.4 (20.5) | -1.48 (-5.30, 2.34)1 |
| **Blood pressure, mm/Hg** |  | |  | |  | |  |  |  |  |
| Systolic | 122.2 (12.9) | | 121.9 (13.7) | | -0.63 (-3.98, 2.72)2 | |  | 119.2 (12.3) | 124.1 (14.8) | 3.92 (-0.51, 8.34)^2^ |
| Diastolic | 74.2 (10.5) | | 75.5 (9.2) | | -0.11 (-2.64, 2.43)^2^ | |  | 74.1 (8.0) | 76.6 (10.2) | 1.84 (-1.22, 4.90)^2^ |
| **Lipids, mmol/l** |  | |  | |  | |  |  |  |  |
| Total cholesterol | 4.0 (0.8) | | 3.8 (0.8) | | -0.26 (-0.48, -0.05)1 | |  | 3.8 (0.9) | 3.6 (0.7) | -0.19 (-0.46, 0.09)1 |
| LDL cholesterol | 2.3 (0.7) | | 2.1 (0.7) | | -0.27 (-0.45, -0.08)1 | |  | 2.1 (0.8) | 2.0 (0.6) | -0.06 (-0.30, 0.17)1 |
| HDL cholesterol | 1.3 (0.4) | | 1.3 (0.4) | | -0.03 (-0.13, 0.07)1 | |  | 1.3 (0.4) | 1.3 (0.4) | -0.04 (-0.17, 0.09)1 |
| Triglycerides | 0.8 (0.3) | | 0.9 (0.5) | | 0.06 (-0.05, 0.17)^1^ | |  | 0.9 (0.4) | 0.8 (0.5) | -0.07 (-0.25, 0.08)1 |
| ART: antiretroviral therapy; BMI: body mass index; LDL: low density lipoprotein; HDL: high density lipoprotein. Obesity defined as BMI≥30kg/m^2^; Elevated blood pressure defined as systolic ≥130 and/or diastolic ≥85 mmHg; Grade 1 hypertension defined as ≥140 and/or ≥90 mmHg. ^1^ Adjusted for: pre-pregnancy BMI, food insecurity status, tuberculosis status, physical activity frequency, age, socioeconomic status. ^2^ Adjusted for: pre-pregnancy BMI, food insecurity status, tuberculosis status, physical activity frequency, socioeconomic status, alcohol use, and age. Missing data: n=5 for BMI, weight, systolic and diastolic blood pressure and n=9 for lipids. | | | | | | | | | | |
| **Table S4. Comparison of characteristics between full cohort and cohort with a postpartum visit.** | | | | | |  |  |  |  |  |
|  | | **Full Cohort** | | **Cohort with postpartum visit** | |  |  |  |  |  |
|  |  | **N=400** | | **N=295** | |  |  |  |  |  |
|  | | **Median (IQR)** | | | |  |  |  |  |  |
| Age | | 30 (25, 34) | | 30 (25, 34) | |  |  |  |  |  |
| Gestational age | | 26 (24, 27) | | 25 (24, 27) | |  |  |  |  |  |
| Parity | | 2.5 (2, 3) | | 3 (2, 3) | |  |  |  |  |  |
| Pre-pregnancy BMI (kg/m^2^) | | 30.1 (25.8, 35.0) | | 31.2 (26.2, 35.8) | |  |  |  |  |  |
|  | | **N (%)** | | | |  |  |  |  |  |
| SES Category | |  | |  | |  |  |  |  |  |
| Lowest | | 129 (32.3) | | 101 (34.2) | |  |  |  |  |  |
| Moderate | | 109 (27.2) | | 77 (26.1) | |  |  |  |  |  |
| Highest | | 162 (40.5) | | 117 (39.7) | |  |  |  |  |  |
| Marital status | |  | |  | |  |  |  |  |  |
| Not married/cohabitating | | 226 (56.6) | | 162 (54.9) | |  |  |  |  |  |
| Married/cohabitating | | 174 (43.5) | | 133 (45.1) | |  |  |  |  |  |
| Primigravida | |  | |  | |  |  |  |  |  |
| No | | 326 (81.5) | | 249 (84.4) | |  |  |  |  |  |
| Yes | | 74 (18.5) | | 46 (15.6) | |  |  |  |  |  |
| Perinatal depression | |  | |  | |  |  |  |  |  |
| No probable depression | | 370 (92.5) | | 273 (92.5) | |  |  |  |  |  |
| Probable depression | | 30 (7.5) | | 22 (7.5) | |  |  |  |  |  |
| Alcohol use | |  | |  | |  |  |  |  |  |
| Below threshold | | 376 (94.0) | | 274 (92.9) | |  |  |  |  |  |
| Hazardous drinking | | 24 (6.0) | | 21 (7.1) | |  |  |  |  |  |
| Food security | |  | |  | |  |  |  |  |  |
| None | | 333 (83.2) | | 236 (80.0) | |  |  |  |  |  |
| Perceived food insecurity | | 67 (16.8) | | 59 (20.0) | |  |  |  |  |  |
| Physical activity | |  | |  | |  |  |  |  |  |
| None | | 181 (45.2) | | 132 (44.8) | |  |  |  |  |  |
| 1-2 times/week | | 100 (25.0) | | 75 (25.4) | |  |  |  |  |  |
| 3-4 times/week | | 93 (23.3) | | 68 (23.0) | |  |  |  |  |  |
| >4 times/week | | 26 (6.5) | | 20 (6.8) | |  |  |  |  |  |
| Family history of diabetes | |  | |  | |  |  |  |  |  |
| No | | 353 (90.0) | | 265 (91.4) | |  |  |  |  |  |
| Yes | | 39 (10.0) | | 25 (8.6) | |  |  |  |  |  |
| Tuberculosis | |  | |  | |  |  |  |  |  |
| No tuberculosis | | 361 (90.2) | | 263 (89.2) | |  |  |  |  |  |
| Previous tuberculosis | | 38 (9.5) | | 31 (10.5) | |  |  |  |  |  |
| Current tuberculosis | | 1 (0.3) | | 1 (0.3) | |  |  |  |  |  |
|  | | **Among participants with HIV** | | | |  |  |  |  |  |
| HIV diagnosis | | **N=200** | | **N=151** | |  |  |  |  |  |
| Before this pregnancy, but during another pregnancy | | 66 (33.0) | | 51 (33.8) | |  |  |  |  |  |
| Before this pregnancy, but not during another pregnancy | | 68 (34.0) | | 53 (35.1) | |  |  |  |  |  |
| During this pregnancy | | 65 (32.5) | | 46 (30.5) | |  |  |  |  |  |
| Perinatally infected | | 1 (0.5) | | 1 (0.7) | |  |  |  |  |  |
| ART initiation | |  | |  | |  |  |  |  |  |
| Pre-conception | | 88 (44.0) | | 67 (44.4) | |  |  |  |  |  |
| Post-conception | | 112 (56.0) | | 84 (55.6) | |  |  |  |  |  |
| ART regimen | |  | |  | |  |  |  |  |  |
| Efavirenz based | | 107 (53.5) | | 74 (49.0) | |  |  |  |  |  |
| Dolutegravir based | | 86 (43.0) | | 71 (47.0) | |  |  |  |  |  |
| Other | | 7 (3.5) | | 6 (4.0) | |  |  |  |  |  |
| Viral load | |  | |  | |  |  |  |  |  |
| Undetectable (<50 copies/ml) | | 62 (69.7) | | 47 (73.4) | |  |  |  |  |  |
| Detectable (≥50 copies/ml) | | 27 (30.3) | | 1 (26.6) | |  |  |  |  |  |
| CD4 count, cells/mm^3^ | |  | |  | |  |  |  |  |  |
| ≤350 | | 47 (29.6) | | 32 (27.1) | |  |  |  |  |  |
| 351 - ≤500 | | 42 (26.4) | | 35 (29.7) | |  |  |  |  |  |
| >500 | | 70 (44.0) | | 51 (43.2) | |  |  |  |  |  |
